# Long-Term Follow-Up of Sexual Dysfunction in Women Following Allogeneic Hematopoietic Stem Cell Transplantation

**DOI:** 10.1007/s10508-018-1296-2

**Published:** 2018-08-21

**Authors:** Katarzyna Skorupska, Tomasz Rechberger, Andrzej Wrobel, Izabela Winkler, Pawel Miotla

**Affiliations:** 1 2nd Department of Gynecology, Medical University in Lublin, Jaczewskiego Street 8, 20-954 Lublin, Poland; 2 St. John’s Cancer Center Lublin II Clinic of Gynecology, Lublin, Poland

**Keywords:** Graft-versus-host disease, Vaginal obliteration, Quality of life

## Abstract

Chronic graft-versus-host disease is the most common late complication following allogeneic hematopoietic stem cell transplantation. The aim of this study was to present the outcomes of two successful vaginal reconstructions. Patient 1 received chemotherapy for leukemia and underwent bone marrow transplantation (BMT). The patient was sexually inactive for 9 years. In 2012, she was diagnosed with complete vaginal obliteration and underwent vaginal reconstruction. Patient 2 underwent chemotherapy (myeloablative therapy), was sexually inactive for 3 years and was then diagnosed with complete vaginal obliteration. In January 2013, she had vaginal reconstruction with cervical dilatation. Hormonal replacement therapy was administered to both patients. The results of dedicated questionnaires revealed decent quality-of-life and normal sexual functioning and continence status after surgery. Obliteration of the vagina after BMT can be prevented, but if it occurs, vaginal reconstruction surgery should be offered to any patients suffering from obliteration. Our results show that this therapy enables patients to have normal sexual lives without compromising their continence status.

## Introduction

Chronic graft-versus-host disease (cGvHD) is the most common late complication following allogeneic hematopoietic stem cell transplantation (allo-HSCT). Allo-HSCT is an intensive therapy used to treat high-risk hematological malignant disorders and other life-threatening diseases. More than 30 conditions are currently treated by hematopoietic cell transplant, resulting in approximately 50,000 transplants a year (Ferrara, Levine, Reddy, & Holler, 2009). Studies show that all patients surviving allo-HSCT between 30 and 90% develop cGvHD, which is associated with decreased quality of life (Sutherland et al., 1997). Data collected from observational studies indicate that between 65 and 85% of all patients with cGvHD have skin manifestations, 60% have mouth involvement, 40–55% have liver involvement, 25–45% have eye involvement, 20–30% have nutritional problems, and 10–15% have lung manifestations (Lee et al., 2002). The incidence of genital GvHD varies from 35% at a 1-year observation after allo-HSCT to 49% at 2 years (Zantomio et al., 2006). According to Stratton et al. (2007), there are three stages of severity of female genital GvHD. The score published by Stratton grades female genital cGvHD on the basis of clinical signs but not symptoms. In Stage 1, the patient suffers from generalized erythema and edema of vulvar structures, patchy erythema of mucosa and glandular structures of vulvar vestibule or erythema around openings of Bartholin’s and Skene’s glands. Stage 2 is characterized by Grade 1 findings plus erosions of mucosal surfaces of the vulva or fissures in vulvar folds. Stage 3 is characterized by Grade 2 findings plus agglutination of clitoral hood, introital stenosis, vaginal synechiae, hematocolpos or complete vaginal closure or fasciitis or spasticity of the levator sling (Frey Tirri et al., 2015).

In this paper, we present the long-term follow-up data collected from two female patients who were suffering from severe genital GvHD, namely the complete obliteration of their vaginas after allo-HSCT, and had successful vaginal reconstruction in our department (the first in 2012, the second in 2013). These operations were previously described and published (Rechberger, Kulik-Rechberger, Winkler, & Perżyło, 2013).

## Case Report Series

### Case 1

A patient, 38 years old with a history of two spontaneous deliveries and no abortions, was admitted to the Gynecology Department in July 2012 due to complete vaginal obliteration. In 2002, she was diagnosed and treated for chronic myelogenous leukemia. She then received 5 chemotherapy cycles (daunorubicin, cytarabine, cladribine and busulfan, cyclophosphamide). In December 2002, she had bone marrow transplantation (BMT), with her brother as the donor. For 1 year after BMT, she received 50 mg of cyclosporine twice a day and 20 mg of methotrexate during the second day after BMT, 15 mg during the fourth day after BMT and 15 mg during the fifteenth day after BMT. The patient declared that her sexual life before leukemia was not satisfying. She got divorced 2 years after BMT. For 9 years after BMT, the patient was sexually inactive due to lack of a partner. In the meantime, she did not seek gynecology consultation, nor was she offered any. However, during that time, the patient had regular oncological consultations. When she found a new partner and tried to have intercourse, it was impossible due to complete vaginal obliteration. According to Stratton, her severity scoring for female genital cGvHD disease was grade 3 (severe) (Stratton et al., 2007). The patient did not have any other manifestations of GvHD. Due to complete vaginal obliteration, conservative management, such as using dilators, could not be used. In July 2012, complete vaginal reconstruction with cervical dilatation was performed under ultrasound control.

The surgical procedure was performed under general anesthesia with the patient placed in the lithotomy position. A Foley catheter was inserted into the bladder to facilitate a proper dissection plane. At the beginning of the procedure, the presumed right plane between the anterior and posterior vaginal walls was identified in the vaginal vestibule. Once the proper plane was identified, careful blunt separation of both fused vaginal walls was performed utilizing the surgeon’s fingers. Superficial bleeding was controlled through bipolar coagulation. Palpation via the urethral catheter placed anteriorly and the insertion of a double-gloved finger along the anterior wall of the rectum posteriorly provided the proper surgical guidelines so that the bladder and rectum could be avoided during this blind procedure. After the dissection was completed, the cervix was visualized, and a Hegar No. 2 dilator was inserted into the cervical canal to confirm its patency. The operation was considered finished once superficial bleeding was stopped and a vaginal phantom (dilator) covered with 0.2% cyclosporine ointment was placed in the vagina.

The vaginal dilator was left in the vagina for 7 days after the surgery, with a 5-day antibiotic prophylactic treatment. Furthermore, on the second day post-operation, 50-µg patches of oestradiol for every 4 days were started. The patient was discharged 7 days after the procedure, with the recommendation to use cyclosporine ointment 0.2% twice a day and the oestradiol ointment-covered vaginal dilator every 3 days. The patient was referred to a sexologist before restarting her sexual activity. Unfortunately, she did not visit a sexologist. One month after surgery, the patient began sexual intercourse. At the beginning, she suffered from minor pain and bleeding. We then offered her estrogen/progestogen oral therapy due to symptoms of estrogen deficiency, but she declined, as she did not want to have any more vaginal bleeding, so she was advised to take continuous hormonal therapy patches containing oestradiol and norethisterone acetate, along with oestradiol vaginal inserts (10 µg) twice a week for 6 weeks. At a 6-week checkup, her vagina had a normal length and capacity, without any narrowing.

### Case 2

A patient, 38 years old with history of one spontaneous delivery and no abortions, was admitted in January 2013 due to complete vaginal obliteration after myeloablative therapy. This patient had been diagnosed with left breast lymphoma in February 2010. She was then given chemotherapy in the Haemato-oncology and Bone Marrow Transplantation Clinic (cytosine arabinoside, daunorubicin, mitoxantrone and epirubicin hydrochloride). Remission-consolidated treatment included further intensive schemes to prevent the recurrence of the disease and five cycles of treatment for maintaining remission. In March 2011, the patient had BMT. In the conditioning treatment in preparation for transplantation, the patient received busulfan, antilymphocyte immunoglobulin and cyclophosphamide. Subsequently, 29 days after BMT, the patient was discharged with a recommendation to use cyclosporine 100 mg twice daily. For almost 3 years, she was sexually inactive, at the beginning due to reduced libido and later due to marital problems. Her sexual life before her health problems was satisfying. The lack of menstruation was attributed to premature ovarian failure (POF) due to the cytotoxic effect of chemotherapy. The patient had regular oncological and psychological consultations. No gynecological consultation was performed in the above-mentioned time frame. When we asked the patient why she did not seek gynecologic consultation in this time period, she said that she was focused on her breast lymphoma treatment and was not thinking about other aspects of her health. Three years after the onset of the disease, this patient asked for a gynecological consultation when she discovered that she was unable to have sexual intercourse due to pain and vaginal narrowing, which she discovered by self-examination of her vagina. During her gynecological vaginal examination, complete vaginal obliteration was found, while no abnormalities were revealed in the sonographic examination. Grade 3 (severe) status for female genital cGvHD disease was assessed according to Stratton’s severity scoring (Stratton et al., 2007). The patient did not have any other manifestations of GvHD. In January 2013, we performed vaginal reconstruction in a manner analogous to that described above. The patient also received cyclosporine ointment and afterward, oestradiol vaginal inserts. Approximately 3 weeks later, the patient arrived for her post-op checkup, and we observed the narrowing of the vaginal walls in the distal 1 cm, which was expanded manually with good visualization of the cervix. We recommended sexological consultation before the initiation of sexual intercourse, which was successfully achieved 2 weeks later without any difficulties. The patient visited the sexologist twice, but she did not feel comfortable during those visits, so she stopped attending. Currently, the patient is on estrogen/progestogen oral therapy due to POF and experiences regular menstruation, while no abnormalities have been revealed in her cervical smear and sonographic findings.

Both patients completed the Short Form (36) Health Survey (SF-36v2), the Female Sexual Function Index (FSFI), the Pelvic Organ Prolapse/Urinary Incontinence Sexual Function Questionnaire (PISQ 12), the Urogenital Distress Inventory (UDI-6) and the Incontinence Impact Questionnaire (IIQ-7) before their surgeries to assess the various aspects of their quality of life (Rogers, Coates, Kammerer-Doak, Khalsa, & Qualls, 2003; Sutherland et al., 1997; Uebersax et al., 1995; Ware & Sherbourne, 1992). Of note, patients with sexual dysfunctions are asked to fulfill FSFI and PISQ 12 as a standard procedure in our department.

Of the aforementioned questionnaires, the SF-36v2 questionnaire is a standardized, validated instrument used to measure health-related quality of life (Ware & Sherbourne, 1992).

The FSFI is a standardized and validated instrument used to measure sexual function. The subscale scores range from 0 to 6, with higher scores indicating better sexual function. The diagnostic cutoff value for diagnosis of female sexual dysfunction for English version of FSFI was calculated as ≤ 26.5 (Wiegel, Meston, & Rosen, 2005). Subjects obtaining a total Polish, validated version of FSFI (PL-FSFI) score of ≤ 27.50 are considered to have sexual dysfunction (Nowosielski, Wróbel, Sioma-Markowska, & Poręba, 2013).

The PISQ-12 is a condition-specific, reliable, validated and self-administered instrument employed to evaluate sexual function in women with pelvic organ prolapse and/or urinary incontinence (Rogers et al., 2003). The PISQ-12 has a range from 0 to 48, with higher scores indicating better sexual function.

The Incontinence Impact Questionnaire (IIQ) measures the impact of urinary incontinence on activities, roles and emotional states, whereas the Urogenital Distress Inventory (UDI) measures how troubling the symptoms are. For both the UDI-6 and the IIQ-7, a higher score indicates higher disability. The results are scored within a 0–400 scale, and the greater the score, the more problematic incontinence is Uebersax et al. (1995).

Four years later (January 2017), both patients were asked to undergo extended gynecological checkups. In the bimanual physical examinations, normal vaginal length, elasticity and capacity were found. To objectively assess these patients’ health, the same questionnaires were used (SF-36v2, FSFI, PISQ 12 and UDI-6, IIQ-7). The questionnaires revealed no troubles with urinary incontinence after surgery, improvement in quality of sexual life and improvement in their mental aspects of general health. There were no changes observed in their physical aspects of life (Table 1). Both patients were sexually active without any compromise due to vaginal obliteration. In addition, both patients used hormonal replacement therapy without any vasomotor symptoms due to POF. Moreover, vaginal sonography revealed no abnormalities.

**Table 1:** Results of the Short Form (36) (SF 36) Health Survey, the Female Sexual Function Index (FSFI), the Pelvic Organ Prolapse/Urinary Incontinence Sexual Function (PISQ-12) and the Urogenital Distress Inventory (UDI-6) and Incontinence Impact Questionnaire (IIQ-7 in both patients

	Categories	Domains	Patient 1: Before surgery	Patient 1: After surgery	Patient 2: Before surgery	Patient 2: After surgery
SF 36	Physical component summary	All	55.92	53.46	56.36	59.13
		Physical functioning	85	85	95	100
		Role-physical	87.5	100	87.5	100
		Bodily pain	100	100	100	100
		General health	42	42	35	90
	Mental component summary	All	38.75	51.42	41.7	59.59
		Vitality	43.75	56.25	56.25	87.5
		Social functioning	75	100	87.5	100
		Role-emotional	91.67	100	83.33	100
		Mental health	40	70	50	95
FSFI	–	Desire	4.2	4.2	4.2	3.6
		Arousal	0	5.1	0	5.4
		Lubrication	0	3.6	0	6.0
		Orgasm	0	4.0	0	6.0
		Satisfaction	0.8	4.8	0.8	6.0
		Pain	0	5.2	0	6.0
		Total	5.0	26.9	5.0	33.0
PISQ-12	–	–	18	36	20	35
UDI-6	–	–	199.8	199.8	66.6	66.6
IIQ-7	–	–	66.6	66.6	0	0

## Discussion

The two patients in this case report showed improvements in their sexual lives (without compromising their continence status) following vaginal reconstruction surgery after obliteration of the vagina due to bone marrow transplantation. The prevention of various permanent dysfunctions among post-transplant patients is of critical importance to enhance the general quality of life after allo-HSCT. To achieve this goal, follow-up programs have been developed that address age- and gender-specific BMT recipients. Although the first description of female genital involvement in cGvHD was published more than 40 years ago (Corson et al., 1982), the problem is still not widely recognized or properly discussed with female hematopoietic SCT recipients. In fact, both health providers and patients tend to neglect genital symptoms with accompanied sexual problems in the early course of the disease, which leads to a significant risk of underdiagnosis of genital cGvHD. Indeed, hematologists, who are focused on the main life-threatening disease, might not specifically inquire about genital symptoms—even if these symptoms are associated with pain, scarring and serious impairment of the patient’s sexual life.

Knowledge of preventive strategy is critical since the early introduction of a proper preventive strategy allows for an avoidance of the majority of genital complications associated with hematopoietic SCT. To achieve this goal, detailed clinical guidelines for gynecologic care after hematopoietic SCT have been developed and published (Frey Tirri et al., 2015). In addition, women should be advised to use vaginal dilators in case of vaginal narrowing or as a prevention of this condition.

Current information holds that oncological diseases pose prominent challenges for patients and their partners. The diagnosis of cancer can significantly change their sexual relationship (Benoot, Saelaert, Hannes, & Bilsen, 2017). Since genital symptoms and sexual impairments are rarely reported spontaneously by patients, it is of pivotal importance that hematologists should ask patients about such symptoms. Furthermore, oncologists or sexologists, who deal with patients after BMT, should know when to refer patients to a gynecologist (i.e., in the presence of any symptoms of genital GvHD or suspicion of human papilloma virus (HPV)-associated cancer, which occurs more often in this group). Additionally, gynecologists should be aware that hormonal therapy is not contraindicated in females after bone marrow transplantation since almost all women after HSCT will experience POF and infertility. Therefore, estrogen/progesterone replacement should be introduced in all women younger than 40 years after initial chemotherapy and HSCT, after assessment of their individual risk for breast cancer and vascular complications. In addition, since a great majority of girls and women after HSCT will be permanently infertile, fertility preservation counseling should be offered.
